# The Dichotomous Responses Driven by β-Defensins

**DOI:** 10.3389/fimmu.2020.01176

**Published:** 2020-06-12

**Authors:** Jennifer R. Shelley, Donald J. Davidson, Julia R. Dorin

**Affiliations:** Centre for Inflammation Research, The University of Edinburgh, Edinburgh BioQuarter, Edinburgh, Scotland

**Keywords:** beta defensin, psoriasis, atopic dermatitis, autoimmunity, immunomodulation, AMP

## Abstract

Defensins are short, rapidly evolving, cationic antimicrobial host defence peptides with a repertoire of functions, still incompletely realised, that extends beyond direct microbial killing. They are released or secreted at epithelial surfaces, and in some cases, from immune cells in response to infection and inflammation. Defensins have been described as endogenous alarmins, alerting the body to danger and responding to inflammatory signals by promoting both local innate and adaptive systemic immune responses. However, there is now increasing evidence that they exert variable control on the response to danger; creating a dichotomous response that can suppress inflammation in some circumstances but exacerbate the response to danger and damage in others and, at higher levels, lead to a cytotoxic effect. Focussing in this review on human β-defensins, we discuss the evidence for their functions as proinflammatory, immune activators amplifying the response to infection or damage signals and/or as mediators of resolution of damage, contributing to a return to homeostasis. Finally, we consider their involvement in the development of autoimmune diseases.

## Introduction

Defensins and defensin-like peptides are found throughout multicellular organisms including plants, insects and fungi, as well as vertebrates. They were first described as antimicrobial peptides (AMP), with the ability to rapidly penetrate and disrupt the outer membrane of bacteria, viruses and fungi to varying degrees and subsequently disrupt metabolic processes within (1). It is appealing to consider that this innate, protective mechanism is so fundamental that the defensin genes have been evolutionarily conserved for this purpose. In fact, the value of the structure of these disulphide-stabilised, cysteine containing, positively charged loop peptides has resulted in two evolutionarily distinct defensin families that have arisen separately by convergent evolution (2). The cis-defensin superfamily (present in insects, fungi and plants), has the central beta-strand stabilised by disulphide bridges, connected to the same alpha-helix in the “cis” orientation. This is in contrast to the vertebrate (and some invertebrate) defensins, in which the central beta strand has disulphide bridges that stabilise structures in non-cis or “trans” orientations (3, 4). Both cis and trans families have undergone rapid expansion and evolutionary change to reveal a repertoire of diverse functions that are only recently becoming clear (5).

Here we focus on the human, trans-defensins—specifically β-defensins. We discuss their role(s) in host defence other than by direct microbial killing. We consider whether the function of these molecules is purely as an acute “alarmin”-type response to danger/damage (alerting the body and promoting both local innate, and also local and systemic adaptive immune responses), or if they are also instrumental in controlling inflammation (limiting the damage response and mediating resolution)—thus speeding a return to homeostasis.

## Beta-Defensin Background

The defensin family is a large, multigene family that is rapidly changing and evolving. In humans there are two functional subfamilies of defensins (α and β) which differ in their cysteine connections but retain the central structure of a trans-defensin cysteine knot. Both α- and β- defensins are generally encoded by two (sometimes three) exons, with the first exon containing the hydrophobic, anionic leader sequence and the second exon encoding the mature, cationic peptide. The α-defensins are stored in this inactive form in the granules of either neutrophils or intestinal Paneth cells, while β-defensins are expressed predominantly in epithelial cells and believed to be cleaved by signal peptidase as they are secreted (6).

β-defensins are an ancient family, from which the α-defensins have evolved. The amino acid sequence of human β-defensins is highly divergent, and has been subject to complex positive and negative selection (7). During this process, other than the cysteines, only a core glycine and aspartic acid are well-conserved. β-Defensins have an identifiable consensus sequence of X_2−10_CX_5−7_(G/A)XCX_3−4_CX_9−13_CX_4−7_CCX_n_ and the three disulphide connections following oxidation are assumed to be the same for all β-defensins (C_I_—C_V_, C_II_—C_IV_, and C_III_—C_VI_). The highly variable residues in the mature peptide are rich in the positively charged amino acids lysine and arginine to varying degrees (7). Some of the peptides have extended peptide tails with clusters of lysines and residues for additional potential glycosylation sites (8). In the human genome there are five β-defensin clusters located over three chromosomes, with around 33 genes, of which only a few have known function (9, 10) and seven of which (in humans) are hyper copy number variable (CNV) (5). The many gene duplications in the defensin family result in gene “birth and death” and as the gene number and sequence changes, some genes become specialised for a new function; while at the species level, there are increased numbers or complete loss of gene clades. Mice, for example, have different numbers of cryptdins (intestinal α-defensins) even between different strains of *Mus domesticus* (11) and no longer have neutrophil expressed α-defensin genes. The sequence diversity and gene number variation in the defensin genes is not surprising as strain specific diverse regions (SSDR) between mouse strains are highly enriched for genes involved in immunity, infection and reproduction functions, all of which are associated with defensins (12).

Gene duplication and sequence change, followed by selection for advantageous changes, allows functional change of paralogues. The structure of some off-shoots of the main β-defensin tree has been so advantageous that there are examples of both reptiles (snakes and lizards) and mammals (egg laying platypus) independently giving rise to venom toxins, with a variety of actions that include antimicrobial function (13) and potassium channel blocking ability (14). Additionally, Kudryashova et al. (15) showed that both α and β-human defensins could target, destabilise and inactivate bacterial protein toxins (16). This work implies that defensins may have protective abilities that are not limited to microbe destruction. Intriguingly, and perhaps indicative of roles of immunological modulation/damage, the human defensin HBD2 has been shown to bind to the outer pore domain of potassium channel Kv1.3 and efficiently inhibit channel currents and suppress IL-2 production in both human primary T cells and peripheral mononuclear cells (17).

At this point, the expression pattern of β-defensins in humans is worthy of mention [*see useful reviews on this here* (18, 19)]. All the many β-defensin members are strongly expressed in various segments of the epididymis post puberty (20, 21) and a major function of β-defensins is in sperm maturation. A β-defensin mutation in human *DEFB126* was found to reduce sperm motility and fertility in Chinese men (22). In addition, mice deleted for several β-defensins (in pairs or more) are infertile and this demonstrates their synergistic function in sperm maturation, movement and protection against premature acrosome reaction (23, 24). Sperm are rich in β-defensin in the glycocalyx of the head and this may protect the sperm from inappropriate activation. However, mice with transgenic over-expression of an epidydimal specific β-defensin (orthologous to human β-defensin SPAG11), while being resistant to *E. coli* infection, simultaneously show reduced expression of inflammatory cytokines IL-1α and IL-1β, indicating multiple functions and implying immunomodulatory properties.

Expression of β-defensins is not just evident in the male genitourinary system, as these peptides are also widely expressed in other tissues. In this review we are focussing primarily on HBD1–4, as these genes are the most studied in human. Their peptide sequence, gene name and charge are given in Table 1. HBD1–3 are found in the female reproductive tract in endometrium, vagina and cervix, while HBD1 is found in fallopian tubes (26). These defensins are increased in expression at a number of sites in the body, including the tracheal epithelium, gingival mucosa, respiratory epithelium, gastrointestinal epithelium, genitourinary tract epithelium and skin (27–30). In addition, HBD1 is produced constitutively in a range of other epithelial tissues, including the small intestine, pancreas, and kidney. Expression of HBD1 may also be increased in various cell types following viral stimulation (31) and both HBD2 and HBD3 are inducible proteins, with expression occurring in various cell types in response to infection (32, 33), proinflammatory cytokines (including IL-1β, IL-17,TNFα, and IL-22) (34–36) and injury. The response to these inducers is not the same for every gene or for every condition. For example, plasma levels of β-defensins are variable in individuals with asthmatic vs. normal airways, where HBD3 is elevated by HBD1 and 2 are reduced (37). HBD3 and HBD4 are significantly increased, but HBD2 is decreased. The level of mouse DEFB14 was also increased in asthmatic animals. Expression may also be varied by genomic copy number of *DEFB103, DEFB4, DEFB104* but *DEFB1* does not show copy number variation (CNV). Both copy number and promoter sequence variation has been shown to contribute to expression of *DEFB4* and *DEFB103* (38, 39), but inflammatory stimuli can override these.

**Table 1:** Mature Peptide sequence of the four human β-defensins described most commonly in the literature.

**PEPTIDE**	**GENE**	**Mature peptide sequence**	**Charge**
HBD1	*DEFB1*	DHYNCVSSGGQCLYSACPIFTKIQGTCYRGKAKCCK	4
HBD2	*DEFB4*	GIGDPVTCLKSGAICHPVFCPRRYKQIGTCGLPGTKCCKKP	6
HBD3	*DEFB103*	GIINTLQKYYCRVRGGRCAVLSCLPKEEQIGKCSTRGRKCCRRKK	11
HBD4	*DEFB104*	ELDRICGYGTARCRKKCRSQEYRIGRCPNTYACCLRKWDESLLNRTKP	7

*Sequences are in single letter code and conserved cysteines are highlighted. DEFB4, DEFB103, and DEFB104 are on the hyper-copy number variable gene cluster in the human genome chr 8p and termed A or B in ensemble to distinguish their independent location in the genome (25). Please note we use here the common peptides names rather than the official designated peptide names (e.g., HBD1 instead of DEFB1etc.). Net charge at pH 7 calculated using https://pepcalc.com/*.

**Figure 1:**
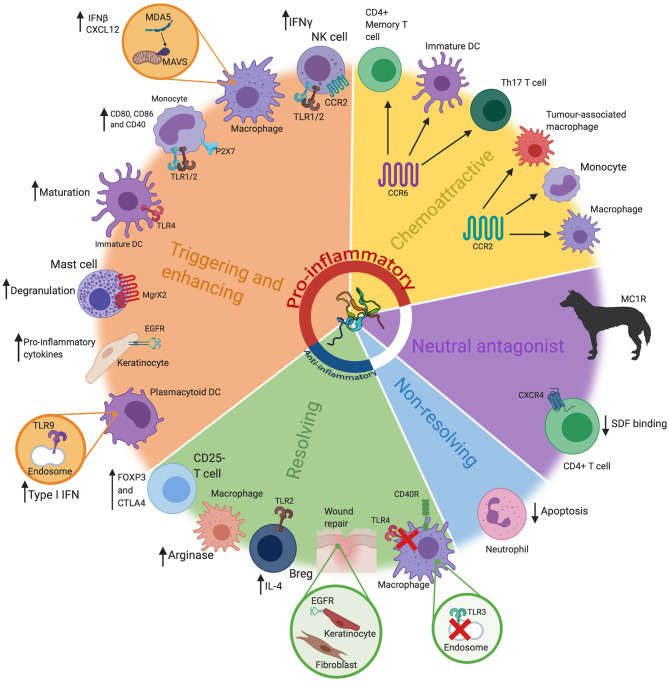
The many roles of β-defensins. β-defensins have been shown to have a wide range of roles, that go far beyond basic antimicrobial activity. These can loosely be grouped into five key groups; triggering and enhancing, chemoattraction (chemoattractive), neutral antagonist, non-resolving and resolving. These functions are represented here, alongside the most prominent cell types/tissues/organisms associated with that particular role. Particular receptors that are known be involved in these pathways have also been highlighted, alongside the consequence of the β-defensin stimulus. These have also been grouped into pro-inflammatory or anti-inflammatory (or neither). Abbreviations: interferon (IFN), toll like receptor (TLR), Dendritic Cell (DC), interleukin (IL), T helper (Th), regulatory B cell (Breg). β*-defensin structure taken from PBD reference 1kj6* *(**44**)*.

This widespread pattern of expression, and inducibility in infection and inflammation, raises the question of what is the principle function of these peptides? A number of studies have been conducted addressing whether β-defensins act as immune or inflammatory modulators, but it is important to bear in mind that synthetic preparation and oxidation of defensins is not trivial. Correct cysteine disulphide bonding and oligomerisation may have an important effect on function as has been shown for the chemoattractive role of defensins (40). In some cases, recombinant peptides have been used, which poses some concern regarding contamination with Lipopolysaccharide (LPS). Some β-defensins are highly charged molecules and their structure *in vivo* can be monomeric or dimeric, oxidised or reduced, depending on the tissue, with known effects on both antimicrobial and other function(s) (40–43). In addition, apart from the reproductive tract, where expression is strong and constitutive, β-defensins are generally expressed at very low levels until induced by inflammatory mediators. The concentration of peptide used in *in vitro* experiments is therefore likely crucial to determine the true *in vivo* effect. Thus, studies using peptides *in vitro* are important, but may not always reflect physiological functional relevance. With those consideration in mind, we discuss below the evidence for β-defensins as host defence peptides, able to modulate the immune system in various ways (see Figure 1).

## Beta-Defensins As Alarmins

Alarmin is a term first coined by Yang and Oppenheim, for endogenous molecules that act as signals for tissue and cell damage (45). They are characterised by a number of central principles, which include the ability to recruit and activate innate immune cells, and bridge to and/or promote adaptive immune responses, whether through direct or indirect mechanisms (46, 47). Increasingly, β-defensins are shown to be involved in pathways of this type, acting as both chemokines for adaptive immune cells and as innate immune stimuli (detailed below). This is suggestive of an alarmin role for these peptides.

### Chemokines

Both β and α-defensins can act as chemoattractants for immune cells (see Figure 1). Some years ago the similarity of defensin structure to chemokines was noted, alongside recognition that many chemokines can have antimicrobial activity under similar experimental conditions to those under which defensins were studied (48, 49). In addition, similarly to chemokines, defensins bind glycosaminoglycans (GAG) and oligomerise (50). Various human β-defensins can attract immune cells including immature dendritic cells, memory CD4^+^ T cells, monocytes, and activated neutrophils at low (~10–100 ng/ml) concentrations, similar to known chemokines (~0.02 μM) (40, 51, 52). When this chemoattractant ability was first described, it was a very exciting observation, revealing defensins as a bridge between the innate response and adaptive immune cell recruitment. CCR6 (receptor for CCL20) was identified as a receptor through which defensins could mediate chemotaxis of lymphocytes and neutrophils, with structural similarities to CCL20 being detected (51–53). However, it was also shown that an as yet unidentified receptor, independent of CCR6, could mediate chemoattraction of CD4+ T cells and dendritic cells by a murine β-defensin (54). In addition, monocytes were shown to be attracted by HBD3 and this activity was shown to be dependent on the cysteine stabilised structure, whereas antibacterial activity was not (40). Interestingly, restoration of a single cysteine (cysV) was sufficient to enable human monocyte chemoattractant activity for HBD3 and its mouse orthologue Defb14 (55). Of physiological relevance, *in vivo* studies only found evidence for the HBD2-mediated attraction of macrophages (and not dendritic cells) following intraperitoneal injection of mice with the peptide (54, 56). Subsequently, CCR2 was shown to be a macrophage receptor through which HBD3 (and Defb14) could induce monocyte/macrophage cell movement (57). Indeed, HBD3 expression has been suggested to result in tumour associated macrophage attraction *in vivo* through CCR2 (58). In addition, HBD1, 2, and 4 can all have their expression increased by ΔNp63 in normal and squamous cell carcinomas and exert a chemotactic activity for (lymphatic) endothelial cells in a CCR6-dependent manner (59).

In addition to these direct chemoattractant properties, defensins can also function indirectly by inducing chemokine expression. Human keratinocytes exposed to a high concentration (30 μg/ml; ~6 μM) of HBD-2, -3, or -4, increase the gene expression and protein production of IL-6, IL-10, IP-10, CCL2, CCL20, and RANTES. The treated cells displayed enhanced Ca^2+^ mobilization, chemoattraction, proliferation and phosphorylation of epidermal growth factor receptor (EGFR); signal transducer and activator of transcription (STAT)1, and STAT3 (60). This pro-inflammatory response was markedly suppressed by G protein coupled receptor inhibitors.

### Innate Triggers

In addition to acting to promote chemotaxis of a range of immune cells, the β-defensins have a range of other modulatory functions that expand their repertoire beyond simplistic microbicidal activity (see Figure 1). A proinflammatory response to HBD3 was observed in monocytes, when a robust concentration of 3.8 μM (20 μg/ml) was used to induce an increase in co-stimulatory molecules CD80, CD86m and CD40 and proinflammatory cytokines in a TLR1/2 dependent manner (61). However, unlike TLR2 ligands, HBD3 did not increase levels of IL-10 and did not reduce co-stimulatory molecule expression (62). At 1 μM (5 μg/ml) HBD3, we see no evidence of proinflammatory responses in primary macrophages. At 5 μM and above, HBD3 can cause membrane damage in monocytes (but not B and T cells) through interaction with the negatively charged phospholipids (63), thus care is required to consider the concentrations at which cellular stress responses to supraphysiological conditions might occur. HBD3-mediated CD86 expression (but not CD80) was shown to be induced via the ATP-gated channel P2X7 (64). Similarly, recombinant mouse β-defensin 2 (*Defb2* peptide) was shown to induce maturation of dendritic cells via TLR4, proposing it as a potential adjuvant, although this was only observed with a fusion protein incorporating this peptide, and not with peptide alone (65).

### Innate Enhancement

In addition to these stimulatory effects of antigen presenting cells, defensins have been shown to alter cellular processing, and inflammatory responses to DNA and RNA. In plasmacytoid dendritic cells (pDC), enhanced intracellular uptake of CpG or self-DNA was observed when the DNA was associated with either HBD3 or HBD2 at a 1:2 μM ratio, thus promoting TLR9-dependent IFN-α production in both human and mouse (66, 67). This was also observed with bacterial DNA in human and mouse pDC, but a response to self-DNA was only seen in the human cells (68). It is likely that these observed increases in ligand uptake and TLR9 signalling are due to the ability of these cell-penetrating peptides to increase the transport of the DNA into the cells (69). However, HBD3 is also able to oligomerise and may increase the ability of the DNA to interact with TLR9 effectively. This has been shown for HBD3 and another cationic host defence peptide cathelicidin, LL-37, as well as other cationic peptides. Schmidt et al. (70) elegantly showed that the peptides can form columnar nanocrystalline complexes with dsDNA and that the distance between the DNA columns influence a stronger or weaker interaction with the TLR9 receptor, which signals to produce type I interferon (71). Importantly these effects have also been observed *in vivo*, with intravenous injection of mice with CpG DNA:HBD3 complexes generating increased IL-6, IFN-γ, IL-12p70, IL-10, and IFN-α in the serum 24 h later when compared to CpG DNA alone, and an increase in antigen presenting cells in the spleen (66).

In addition, primary mouse macrophages, when pre-stimulated for 4 h with a fusion protein of IgG1 and the mouse orthologue of HBD3, Defb14, then subsequently stimulated for 24 h with endosomal (TLR3 and TLR9) or heterodimer (TLR1/2) ligands, showed an increase in proinflammatory cytokines and chemokine CXCL12, independent of the presence of CCR2 or CCR6 (72). The Defb14 fusion did not induce a cytokine signal on its own. These studies reveal a complex interplay with other factors, via which these defensins may contribute to enhanced adaptive responses.

Our lab has shown that the presence of HBD3 alters innate signalling to double stranded RNA poly I:C, increasing the Interferon-β (IFNβ) response and decreasing CXCL10 (IP10) production *in vivo* and *in vitro* in both mouse and human primary macrophages (73). PolyI:C is a synthetic double stranded RNA (dsRNA) and consequently acts as a mimic of virus or product of damaged cells. It is recognised by endosomally located TLR3 and also by cytoplasmic RIG-I-like receptors (RLRs). High molecular weight (HMW) poly I:C preferentially signals through the RLR MDA5 (Melanoma Differentiation-Associated protein 5), also known as IFIH1 (interferon induced with helicase C domain 1) and produces Interferon β (IFNβ). We showed that 0.1 μM HBD3 enhanced poly I:C-mediated MAVS (IPS-1) and MDA5 signalling, increasing IFNβ, but decreased TLR3 stimulation and CXCL10 signalling (72) in primary murine macrophages. The peptide rapidly entered the macrophages (within 10 min), decreased the endosomal localisation of the HMW PolyI:C and increased cytoplasmic localisation. This contrasted with the effect of the cationic lipid lipofectamine on HMW PolyI:C, which increased endosomal signalling through TLR3. LL-37, a cathelicidin cationic AMP with some similar immunomodulatory actions to HBD3 (74), can also increase dsRNA induced signalling through MAVS and TLR3 to increase production of proinflammatory cytokines and IFNβ in keratinocytes (75). For TLR3 this is partially due to the alpha helical LL-37 peptide forming crystalline structures with dsRNA which matches the steric size of TLR3, allowing recruitment and engagement of multiple TLR3 receptors and an increased cytokine signalling response (76) in a similar way to peptide-induced DNA association with TLR9. The increased signalling by MDA5 in the presence of HBD3 and HMW polyI:C might also be structurally dependent. Of note, linear HBD3 peptide does not increase IFNβ production and MDA5 normally forms filaments around dsRNA for oligomerization; we therefore speculate that this may be optimised in the presence of correctly-folded HBD3 (77).

The properties of other immune cells can also be modified by exposure to defensins, to promote host defence mechanisms. In the presence of HBD3, human NK cells increase CD69 C-Type lectin protein expression and secrete IFNγ, killing the NK sensitive myeloid cell line K562. In addition, HBD3 can function through the Mas related gene X2 to activate and initiate degranulation of mast cells (78, 79). Other cationic amphipathic peptides, such as LL-37, have also been shown to have this capacity.

Finally, defensins may modulate cell death, with possible consequences for inflammation. β-defensins have been shown to downregulate the pro-apoptotic truncated protein Bid and upregulate the anti-apoptotic Bcl-xL, leading to inhibition of mitochondrial membrane potential change and decreased caspase 3 activity and apoptosis (80). HBD3 is the most potent of the human β-defensins in this regard. Neutrophil apoptosis is important in resolution of tissue damage, thus limiting apoptosis may also be pro-inflammatory. In contrast, in human airway smooth muscle cells, the addition of HBD3 (at high concentrations of 5 or 10 μM) has been shown to induce CCR6-dependent production of IL-8 and cell apoptosis. This apoptotic effect appeared to be induced by ERK1/2 MAPK and ROS-induction (37). This may be important context for scenarios in which higher concentrations of the peptide are seen to be inflammatory and leading to cytotoxic effects. Cytotoxicity has been seen for high concentrations of HBD3 (over 20 μM) in a wide variety of cells in culture, including DC, normal and immortalised keratinocytes and primary oral gingival epithelial cells (81).

### Receptor Neutral Antagonism

There are several examples of defensins acting as promiscuous ligands for receptors (see Figure 1) helping to explain the pleiotropic properties observed. This may be due to complementary electrostatic interaction between the cationic peptide and receptors with anionic regions. HBD3, the most highly charged β-defensin (charge of +11) has been demonstrated to be a neutral antagonist, through charge based interaction with melanocortin receptor 1 and 4 (82). In dogs, a three base pair deletion in the canine orthologue of HBD3 results in an increase in the level of expression, which then allows this peptide to promiscuously bind the melanocortin receptor 1 (MC1R)—resulting in dogs with black, rather than agouti, fur (83). When the mutant or wildtype dog genes are expressed ubiquitously in transgenic mice, under a powerful promoter, their coat colour is also black (despite being genetically agouti). This demonstrates that an inappropriately high, level of β-defensin can result in promiscuous receptor binding *in vivo*. A further example of promiscuous receptor binding and neutral antagonist behaviour is the ability of recombinant HBD3 (at 5, 10, 20, and 40 μg/ml) to compete with stromal-derived factor 1 (SDF-1), in a structural and charge dependent manner, for cellular binding to CXCR4, without increasing calcium mobilization or chemotaxis (84, 85). CXCR4, also known as fusin, is used for HIV entry into CD4+ T cells. However, copy number increase of the HBD3 gene does not associate with protection against HIV (86).

## Beta-Defensins As Resolvers

In contrast to alarmin activity (see section Beta-Defensins as Alarmins) we use the term “resolvers” here to describe the anti-inflammatory pro-resolving activity of β-defensins.

### Innate Suppression

As discussed above, in the presence of defensins, some pattern recognition receptors increase the response to stimulation. However, exposure to TLR4 ligands (such as LPS) or CD40 activation in the presence of HBD3 (1 μM) results in a decrease in cytokine responses in primary macrophages (87, 88). This anti-inflammatory effect was also observed *in vivo*, where serum from mice displayed a decrease in proinflammatory cytokines following injection of LPS and HBD3 peptide compared to LPS alone (87). This suppression was independent of defensin binding to TLR4 or LPS and could be observed even if the peptide was added up to an hour after the LPS. HBD3 suppressed cytokine and type I interferon production through the MyD88 and TICAM1 pathways, respectively (89). Exposure to HBD3 and LPS compared to LPS alone showed reduced transcription of many genes associated with TLR4 activation, while others were increased, including TLR2—demonstrating that this was not simply an inhibition of all signalling downstream of the receptor. HBD3 alone had no effect on macrophage transcription. Further pathway analysis, using InnateDB, showed that many LPS-induced proinflammatory signalling pathways were downregulated when HBD3 was also present but that metabolism, classical complement activation and FcγR-dependent phagocytosis were upregulated (74). The anti-inflammatory effect of HBD3 on macrophages was also seen in the acute inflammatory cytokine response to *Porphyromonas gingivalis in vitro* and *in vivo* (88). Indeed, mice with an exaggerated response to *P. gingivalis* LPS (*ApoE –*/–) showed an increase in CCL2, TNF-α, IL-6, and NO levels at 2 h—but HBD3 (10 μg/mouse) could suppress this. The authors also report an increase in *Arginase 1*, a key marker of mouse alternatively activated macrophages (AMM or termed M2), possibly indicating a change in cellular polarisation as a consequence of defensin exposure.

A similar inflammation suppressive effect has also been recently observed with HBD2, which reduced TNFα and IL-1β secretion from dendritic cells in human peripheral blood mononuclear cells exposed to LPS. The effect was lost in the presence of a CCR2 inhibitor. When HBD2 was delivered systemically to a variety of mouse models of inflammatory bowel disease, the colitis was reduced to a level comparable to steroids and anti-TNFα (90). In addition, in the infected cornea of mice, silencing of the murine orthologues of HBD2 and 3 resulted in increased production of proinflammatory cytokines, with a simultaneous increase in bacterial load (91). The effect on bacterial load is postulated to be due to the defensins [at low concentration of 1 μg/ml (0.2 μM)] inhibiting macrophage autophagy and in this way increasing phagocytic receptor expression leading to intracellular killing of the *Pseudomonas aeruginosa* (92). All these studies indicate that, under specific infections scenarios, defensins are capable of contributing to anti-inflammatory response, or at least a rebalancing of the nature of the cellular response.

### Adaptive Suppression

In addition to effects on innate responses to infectious and inflammatory stimuli, defensins have also been shown to have suppressive effects on adaptive immunity. UVB radiation induces Defb14 production in keratinocytes while DEFB14 peptide injection into mice suppressed contact hypersensitivity, but this was shown to involve the induction of antigen-specific regulatory T cells (Tregs), rather than the UV suppression pathway (93). The HBD3 peptide [at 10 μg/ml (2 μM)] has a demonstrated capacity to alter CD4+ CD25- T cells, from a non-regulatory phenotype towards a regulatory phenotype with expression of both the characteristic regulatory T cell (Treg) transcription factor (*FoxP3*) and cytotoxic T-lymphocyte-associated protein 4 (CTLA4), a protein which downregulates immune responses (94). Treatment of CD4+CD25– cells with DEFB14 resulted in reduction in methylation of the *Foxp3* promoter compared to cells without DEFB14 (and closer to the level seen in Tregs) which correlated with an increase in FoxP3 expression. Additionally, treatment with DEFB14 before, or after, the induction of experimental autoimmune encephalomyelitis (mouse model of multiple sclerosis), was found to ameliorate the disease, with less central nervous system inflammation and decreased levels of proinflammatory cytokines and cytotoxic T cells (94). The beneficial effect was lost upon depletion of regulatory T cells. These observations were attributable to an increase in suppressive CD4+ T cells, possibly through a change in cellular polarisation but the mechanism underpinning defensin-induced modulation of CD4+ T cells to a regulatory phenotype requires further investigation.

Further evidence of immune suppression by HBD3 (or DEFB14) arises from studies of diabetes. β-defensins were shown to be expressed in endocrine cells in both the human and mouse pancreas (95). DEFB14 treatment of non-obese diabetic mice was found to dampen the autoimmune response and to reduce subsequent diabetes development. This disease limitation was shown to be due to DEFB14 increasing proliferation of pancreatic B cells, expressing the regulatory cytokine IL-4 and the repair cytokine active TGFβ, which enabled polarisation of alternatively activated macrophages and a subsequent increase in Treg cells. This immune modulating pathway was believed to account for the reduction in autoimmune inflammation, with DEFB14 playing an integral role possibly through induction of TLR2.

### Wound Healing Resolution

In addition to roles in innate and adaptive immune responses, β-defensins have been found to play important roles in resolution of damage pathways, via effects on wound healing. Characterising chronic wounds, β-defensin expression is found to be decreased in diabetic ulcers (96). This is thought to contribute to increased infection and also to a lack of wound healing, through mechanisms such as stimulating the migration of fibroblasts, as well as the proliferation of keratinocytes (97, 98). HBD2 also is reported to promote wound healing of intestinal cells *in vitro* (99) and *in vivo* by stimulating keratinocyte migration and proliferation in rats (100). The physiological significance of these findings are demonstrated in mice with *Defb14* deletion, which display delayed wound healing *in vivo*, with significantly increased wound area, delayed epithelialisation and an altered wound microbiota (97). In addition, there is an observed increase in classically activated macrophages in these wound sites and a trend towards decreased alternatively activated macrophages, together with an increased bacterial load in the skin (97). This implies that DEFB14 is important in wound repair and that insufficient peptide expression may reduce wound healing as a consequence of inappropriate macrophage polarisation (section Innate Suppression) and/or alteration in the ratio of local cellular populations. Macrophages are key in wound repair and can be central in the process by promoting a resolution of inflammation leading to tissue repair. CCL2, the major macrophage chemoattractant, can reverse the impaired wound healing in diabetic mice (101) and HBD3 can chemoattract macrophages through CCR2 and also modulate pattern recognition receptors relevant to wound repair (102, 103). As with all resolution milieu, successful wound healing will be multifactorial, but these data suggest that β-defensins are likely to contribute.

## Human Disease Association

Given the range of roles that β-defensins display, it is not surprising that their expression and influence are demonstrably intertwined into various disease states. For the sake of examining β-defensins in human non-infectious disease, however, discussion will be based on the main expression sites, epithelial cells, predominantly localised to the gut and skin. We will not be addressing their influence on cancer, although the involvement of β-defensins in cancer also demonstrates dichotomous behaviour. For example, their expression can be increased or decreased in tumours, their influence can be to promote or suppress, and these effects can be dependent upon the specific defensin peptide, the cancer type and the cells involved [for an excellent, recent review of the literature see (104)].

In addition to the complex, localised environmental influences that dictate β-defensin function, there is the issue of copy number variation, as mentioned previously. Six β-defensin genes (*DEFB4, DEFB103, DEFB104, DEFB105, DEFB106, DEFB107*), and the β-defensin related gene *SPAG11*, are present at chromosome 8p23.1, at two loci 5 Mb apart and are hyper CNV, changing through unequal crossing over at the rate of ~0.7% per gamete (25). Worldwide, the average copy number of this unit is four, although copy numbers range from two to twelve (this does not occur in all species, with mice being an example of no copy number variation). The variation in these genes, combined with the alteration in expression based on localised stimulation, gives a large range of expression for these peptides, with overall inflammation in disease considered a stronger influence on expression than copy number (5, 105). The link between the level of expression of β-defensins and disease is discussed below.

### Psoriasis

**Figure 2:**
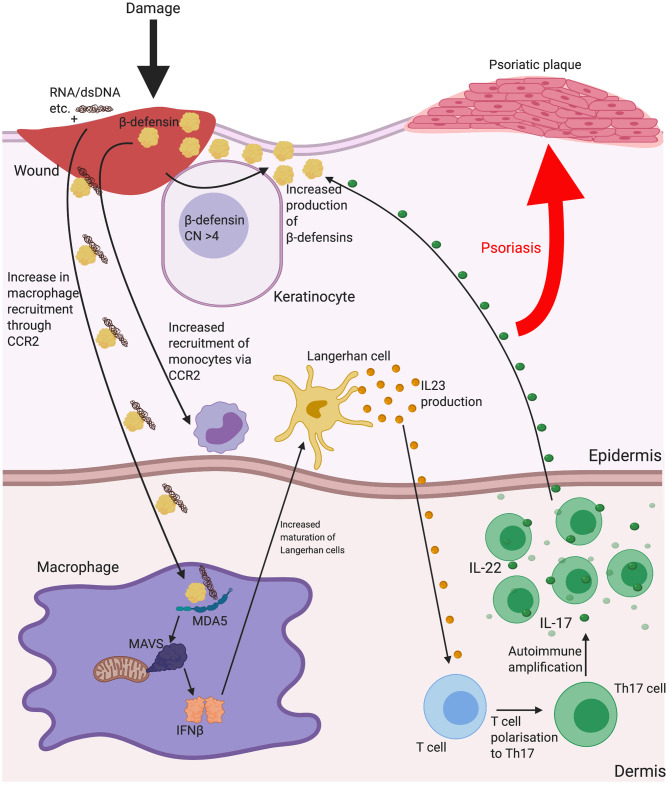
The possible implications for β-defensins in psoriasis. Psoriasis is a disease characterised by scaly lesions, hyperplastic epidermal thickening, immune cell accumulation and is triggered by some sort of insult to the skin. HBD2 and 3 may contribute to the disease process as a consequence of increased gene copy number increasing the level of the peptide response to inflammation and enhancing monocyte/macrophage recruitment and increasing uptake of nucleic acids released from dying cells or microbes at the site of damage. Shown here is increased dsRNA entering macrophages with β-defensin and enhancing IFNβ secretion leading to Langerhan cell maturation and interleukin (IL)-23 release to influence mature T cell polarisation to T helper (Th)17. IL-22 production from Th17 may then further stimulate β-defensin production and amplify the process. Abbreviations: Copy number (CN), C-C chemokine receptor type 2 (CCR2), interleukin (IL), melanoma differentiation-associated protein 5 (MDA5), T helper (Th).

Indication that β-defensin copy number associates with disease development is evident in psoriasis. Psoriasis is a disease principally characterized by skin plaques, commonly found on the elbows, knees and trunk. Psoriatic lesions are described as sites of chronic skin inflammation with thickened, hyperplastic epidermis, increased vascularity and immune cell invasion. Lesions display overexpression of several inflammatory peptides and cytokines. The overexpression of these local cytokines (such as TNFα, IFNγ and IL-1) leads to increased expression of β-defensins within the lesions (106, 107), to a degree that allowed both HBD2 and HBD3 to be first isolated from psoriatic scales. In addition, there is a significant, replicated association between more than four copy numbers of the β-defensin seven gene repeat unit and psoriasis occurrence (108, 109). This goes beyond localised disease region expression, however, as serum levels of HBD2 correlate with copy number, both in normal individuals and disease state (where increase in serum HBD2 also correlates with psoriasis severity) (110) and HBD3 expression is increased in both normal and lesional skin of psoriasis patients, possibly adding to the reduced bacterial burdens in lesions compared with those in atopic dermatitis (see below) (111). It is not clear how increased β-defensin expression contributes to disease aetiology, but, as mentioned above, β-defensins HBD3 and 2 have been shown to increase the Interferon-α response to DNA via TLR9 and the Interferon-β response to RNA through stimulation of the MDA5/MAVS pathway (see section Innate Triggers). HBD2 has been shown to be evident in the same dermal compartment as pDC in psoriatic skin, leading to a hypothesis that this peptide may be instrumental in breaking tolerance to self-DNA following infection or damage (67). Injection of CpG DNA**:**HBD3-defensin complexes subcutaneously in mice, increased epidermal hyperplasia and both neutrophil and lymphocyte recruitment at 24 h (66), supporting this hypothesis. Psoriasis is a Th17 predominated disease and effective treatment with UV irradiation is linked to suppression of type I Interferon and Th17 cells (112). In addition, psoriasis can be induced in multiple sclerosis patients using IFNβ therapy (113). The current effective treatments for psoriasis are biologics against IL-17 production or IL-12p40 (subunit common to both Il-12 and IL-23), to limit Th17 cell production and action. Of note, IL-22 is expressed by Th17 cells, which triggers β-defensin expression (95). Interestingly, individuals with missense variants in Human MDA5 gene (*IFIH1*) are protected from psoriasis (114) and gain of function MDA5 mutations have related type I interferonopathy with musculoskeletal disease that includes psoriasis (115). These lines of evidence strongly support the involvement of Interferon β in psoriasis and the genetic link between increased β-defensin CNV and psoriasis may be due to an increase in β-defensin expression having a functional consequence in the responses to dsRNA released from wounds, via MDA5/MAVS signalling and production of IFNβ (see Figure 2).

### Atopic Dermatitis

**Figure 3:**
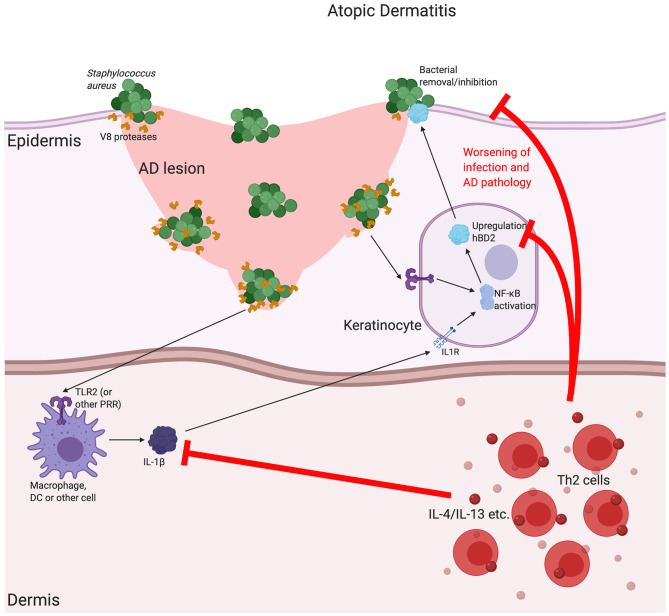
The possible implications for β-defensins in atopic dermatitis. Atopic dermatitis (AD) is a chronic, relapsing disease associated with itchy lesions on the skin, across a large proportion of the body. These lesions are characterised by a breakdown in the barrier function of the uppermost regions of the skin (the epidermis). This allows for an increase in bacterial infection, which is made worse by bacterial production of proteases that further breakdown the junctions between cells, as is the case for V8 (SspA) production by *Staphylococcus aureus*. In AD, there is a downregulation in a number of Th1-associated cytokines, such as Tumour Necrosis Factor (TNF)α and interleukin (IL)-1β, as well as antimicrobial peptides, such as the β-defensins (including HBD2). It is thought that the AD-associated, localised cytokine milieu, which has a T helper (Th)2-skewed phenotype, is responsible for this reduction. Inhibition of the induction of β-defensins prevents proper bacterial removal/inhibition, worsening infection and AD pathology. Abbreviations: interleukin (IL), Toll Like Receptor (TLR), pathogen recognition receptor (PRR).

Another skin disease associated with β-defensin expression is atopic dermatitis (AD) [for a more extensive review of the associations between β-defensin and AD see Chieosilapatham et al. (116)]. AD is another chronic inflammatory skin disease, characterised by itchy, inflamed lesions across a range of different body sites (117). In comparison with psoriatic plaques, AD lesions have a decrease in expression of these β-defensins (118) with induction of peptide levels found to be impaired for the level of inflammation. This has not been found to be related to copy number variation, however, and is instead due to the local Th2-skewed cytokine milieu and thus focused inhibition of β-defensin expression (119). Despite these lesional differences, HBD2, but not HBD3, is found to be increased in AD serum (120). Reduced defensins at the sites of disease may contribute to the pathology of AD in a number of ways, including the increase in lesional skin infections that are characteristic of the condition (121) (see Figure 3). In addition to the direct bactericidal properties of some of these peptides, it has been shown that HBD3 can increase expression of tight junction components in keratinocytes and improve barrier function (122). Further, we recently demonstrated that some β-defensins, such as HBD2, are able to inhibit the barrier-damaging effects of bacterial proteases, such as from the common AD lesional pathogen *Staphylococcus aureus*, which can contribute to this disease, in which loss of barrier integrity is critical (123). The mechanism of this is not yet fully elucidated and is subject to further investigation.

### Inflammatory Bowel Disease

In addition to inflammatory disorders of the skin, β-defensin expression has been shown to be altered in chronic inflammatory disorders of the gut. Unlike the skin, however, β-defensins are not the key AMP type in the gastrointestinal tract. Instead, the most abundantly expressed AMP group is the human α-defensins, including human defensin 5 (HD5) and human defensin 6 (HD6), which are constitutively expressed by Paneth cells located at the base of the crypts of Luberkühn (unique to the small intestine) (124, 125). As well as being known to have antibacterial (HD5) and antiviral (HD6) activities (126), these peptides are known to be chemoattractive for naïve and memory CD4+ T lymphocytes, as well as macrophages and mast cells (127). Similarly to their β-defensin cousins, they are also linked chronic inflammatory disorders, with decreased levels of both HD5 and HD6 being demonstrated in ileal Crohn's disease (affecting the upper parts of the intestine) (128). It is thought that this lack of expression allows for increased pathogenic bacteria and therefore worsening of pathology (129).

While α-defensin are present in Paneth cell granules in the upper parts of the intestine, β-defensin expression is conducted by enterocytes, which are the most abundant epithelial cell lineage in both the small and large intestine (130). Enterocytes of the colon express HBD1 constitutively, with HBD2 being induced by TLR stimulation (131). HBDs can also be induced in the gastric mucosa, when faced with bacterial challenge (132) and expression of β-defensins is shown to be altered in chronic inflammatory bowel diseases (IBD) of the gut. Comparably to the relationship between β-defensin levels and different chronic inflammatory disorders of the skin, there appears to be a discrepancy in activity in different IBD disorders of the gut. Patients who suffer from Crohn's disease present a decrease in HBD2 β-defensin levels, and a concomitant decrease in the gene copy repeating unit suggested as a factor for predisposition to the disease (133, 134). HBD2 has recently been delivered subcutaneously to mice with induced models of intestinal bowel disease and successfully reduced the level of inflammation (90). In opposition to Crohn's disease, patients with ulcerative colitis (a disease of the colon) have a highly increased expression of HBD2, although not HBD1 (135). This has been argued to be due to changes in localised cytokine milieu, rather than variations in copy number. Aldhous et al. demonstrate that *DEFB4* mRNA and HBD2 protein levels varied upon stimulation with inflammatory cytokines in samples from IBD patients, independent of variations in HBD2 copy number (105). In this case, the influence of copy number variation is overridden by the impact of the local inflammatory environment. However, this is in the context of the high level of variation in defensin expression from one region of the gut to another, which is also in combination with differences between biopsy location and inflamed vs. non-inflamed areas of the bowel (105). This requires further study and the influence of the microbiota on defensin expression and defensin expression on microbiota composition needs clarification.

## Conclusion

It is clear that β-defensins are not only AMPs, and their ability to change the behaviour of eukaryotic (particularly immune) cells at similar concentrations as those required to kill pathogens is intriguing. Here we have described the increasing body of research that has revealed the ability of β-defensins to behave in a dichotomous way with respect to inflammation. Under certain conditions they behave as alarmins and yet under other conditions they are suppressors of inflammation. The difference in effect does not seem to be due to the levels of peptide. As the Dorin lab has shown, the same peptide preparation on human and mouse primary macrophages can suppress or increase inflammatory signalling, dependent on which PRR ligand is used. The charged nature of β-defensins is likely to be important in how it interacts with a variety of molecules and may explain why HBD3, with its high charge, consistently gives the most potent responses. Blocking receptors, binding to nucleic acids to enhance receptor engagement, and inducing chemoattraction, are all likely to be driven by the cationic and amphipathic nature of the peptides. At higher levels (above 2 μM) β-defensins certainly have a cytotoxic effect, but this may be supraphysiological. During infection, rapid killing, detection and innate response are essential; therefore, in this regard, high HBD3 copy number and potentiation of PRR may be beneficial. However, an undesirable effect of increased copy number of the defensin cluster (and concomitant increase in expression of defensin peptides) may be over stimulation of PRRs leading to exuberant production of type I interferons. This double-edged sword may provide protection against pathogens in the short term, but in the longer term contribute to the development of psoriasis in individuals with an increased copy number of the β-defensin cluster.

*In vivo* experiments are the most compelling to attribute function, because other cationic host defence peptides will also play a part, as some have synergistic actions and come from recruited, as well as resident cells at the site of injury. The *in vivo* evidence that DEFB14 or HBD3 can increase the inflammatory state of mouse skin but increase wound healing and suppress development of autoimmune diabetes are clear demonstrations of the dichotomy of the influence of β-defensins on mammalian cells. The influence of increased β-defensin expression in psoriasis and reduced expression in atopic dermatitis may reflect the different disease environments; in this case increased copy number in psoriasis may be the causative factor. This is an exciting area of research and further clarification of the factors that give rise to the type of response β-defensins encourage is important for therapeutic strategies.

## Author's Note

We apologize to our colleagues whose work we were unable to cite due to space limitation.

## Author Contributions

Manuscript conceived, researched and written by JS and JD, with additional interpretation, writing and editing by DD.

## Conflict of Interest

The authors declare that the research was conducted in the absence of any commercial or financial relationships that could be construed as a potential conflict of interest.
